# Pulsed Radiofrequency-Enhanced Dual Sympathetic Block for the Treatment of Post-Traumatic Stress Disorder

**DOI:** 10.7759/cureus.41309

**Published:** 2023-07-03

**Authors:** Tabitha Block, Jonathann Kuo, Marcel Green

**Affiliations:** 1 Research, Hudson Medical Group, New York, USA; 2 Regenerative and Anti-Aging Medicine, Hudson Medical Group, New York, USA; 3 Psychiatry, Hudson Medical Group, New York, USA

**Keywords:** post traumatic stress disorder, pulsed radiofrequency ablation, pulsed radiofrequency, post traumatic stress disorder (ptsd), ptsd symptoms, stellate ganglion block (sgb)

## Abstract

Dual sympathetic blocks (DSBs) have been shown to provide significant symptom relief in individuals with post-traumatic stress disorder (PTSD). However, despite the clinical significance of DSB in PTSD treatment, a subset of patients experience the recurrence of somatic symptoms of PTSD and trauma-induced anxiety. The purpose of this case report is to describe our experience with the successful treatment of acute symptoms of PTSD by using serial DSBs and DSBs with pulsed radiofrequency (PRF).

An 18-year-old male who had suffered multiple childhood traumatic events presented with severe and persistent symptoms consistent with a diagnosis of PTSD. The patient had been previously treated with myriad, multiple-year trials of psychotropic medications and psychotherapy as well as lifestyle modifications involving art therapy and physical exercise. Despite these psychiatric and psychological interventions, his symptoms persisted. The patient underwent a total of four bilateral DSBs, three of which were enhanced with PRF, over a period of 15 months at our clinic, with intervals of three, four, and six months between appointments, respectively. At the two-week follow-up after the initial bilateral stellate ganglion block (SGB) procedure, a major improvement in the patient's PTSD symptoms was observed, specifically symptoms of anxiety and a heightened sense of danger. These results were confirmed by a reduction in PTSD Checklist Version 5 (PCL-5) scores from 73 to 50. However, three months later, some of his symptoms returned. The patient elected to proceed with a bilateral PRF-enhanced DSB, and he subsequently reported that his PTSD and general anxiety symptoms subsided by 80%, which was confirmed by a reduction in PCL-5 scores from 50 to 42. This significant symptom relief persisted for four months, and the patient returned for his second bilateral PRF-enhanced DSB. The patient’s PCL-5 score further dropped from 42 to 22 and he reported an 80% reduction in symptoms, which persisted for six months. The patient elected to undergo a third bilateral PRF-enhanced DSB, which was successful in further reducing his symptoms as demonstrated by a self-reported 80% symptom relief and a drop in PCL-5 scores from 22 to 20, which has persisted for over six months.

We highlight the fact that the addition of PRF in a selective blockade of the stellate ganglion via injection reduced our patient’s PTSD symptoms to below the PTSD diagnostic threshold. Furthermore, we report that the clinical efficacy of bilateral PRF-enhanced DSB may be additive over successive procedures. We also provide a theoretical exposition of our findings.

## Introduction

Post-traumatic stress disorder (PTSD) is a disabling psychiatric disorder associated with functional and cognitive impairments that result from exposure to real or perceived physical or mental injury or threat [1]. The Diagnostic and Statistical Manual of Mental Disorders, Fifth Edition (DSM-5) defines PTSD symptoms as belonging to one or more of the following four categories: intrusion, avoidance (of thoughts and behaviors), negative changes in thoughts and mood, changes in arousal (and reactivity) [1]. PTSD is characterized by re-experience and avoidance symptoms such as intrusive thoughts, nightmares, flashbacks, dissociation, intense negative emotions, problems with sleep and concentration, irritability, increased reactivity, increased startle response, and hypervigilance [1]. As PTSD is one of the strongest correlates of suicidal ideation, lifetime suicide plans, and suicide attempts, it has profound implications in terms of individual and global health levels [2-3]. PTSD can significantly impair individual, social, and family functioning, and high rates of PTSD comorbidity with depression, substance abuse disorders, and physical health problems ultimately result in poor individual-level outcomes [1]. While first-line PTSD treatment protocols typically include trauma-focused cognitive behavior therapy in combination with pharmacological interventions, only a subset of patients find these treatments adequate for symptom relief or even symptom remission, while many patients with PTSD continue to struggle to manage their symptoms despite seeking care. Surveys of individuals with PTSD have revealed numerous limitations to these conventional methods, including concerns about emotional readiness for treatment, stigma, and logistical issues [4]. Similarly, many traditional pharmaceutical interventions have a slow onset of action and can pose serious side effects, leading to high rates of poor compliance with such treatment protocols [4].

Stellate ganglion blocks (SGBs) have been recently demonstrated to provide significant and long-lasting PTSD symptom relief, including improvements in anxiety, negative mood, and hyperarousal [5-10]. SGB is a specialized sympathetic nerve block in which a local anesthetic such as bupivacaine is injected into the region of the cervical sympathetic ganglion. The procedures are typically repeated on the contralateral neck to enhance their therapeutic potential. When used in conjunction with trauma-focused psychotherapy, SGBs have been shown to have a 70-80% success rate in treating PTSD symptoms [5-10]. Furthermore, there is a significant evidence base indicating that SGB may provide durable and lasting symptom relief for at least a subset of PTSD patients. A 2014 clinical case study involving 166 active duty service members reported that of the 166 patients who received a right-sided SGB, 70% had a clinically significant improvement in PTSD symptoms, which persisted beyond three to six months post-procedure [7]. Additionally, SGB can be administered as part of a bilateral series over two appointments to enhance the efficacy of a single-sided SGB, and this set of procedures is called dual sympathetic blocks (DSBs) [9]. However, despite the high success rate and strong safety profile of SGB and DSB for PTSD, there is a subset of patients who, while experiencing a significant improvement in symptoms initially, may continue to suffer from the condition. Implementing modifications to the traditional SGB procedure that extend the length of its clinical benefits would have profound implications for this subset of PTSD patients.

In this case report, we discuss sustained positive clinical outcomes observed in one PTSD patient following treatment with a cervical sympathetic blockade at the C6 level followed by the C4 level targeting superior cervical ganglion, thereby displaying the enhanced efficacy of DSB with the addition of pulsed radiofrequency (PRF) at C6 and C4 levels in at least a subset of PTSD patients.

## Case presentation

The patient was an 18-year-old male who had suffered multiple childhood traumatic events beginning at age two when his mother had been diagnosed with cancer and he had been left in the care of his grandfather, after which he had felt neglected and abandoned. At age three, the patient had been in a car accident in which family members had been injured. From kindergarten to 7th grade, the patient had experienced recurrent trauma in the school system during “active shooter” drills and had been eventually withdrawn from school to be homeschooled. The patient had begun using a therapy dog at eight years of age to help manage his symptoms of severe anxiety and a heightened sense of being attacked. Three years before presenting to our clinic, the patient had tried to resuscitate his therapy dog after it had a stroke. The patient reported that this traumatic event motivated him to seek a psychiatric and psychological assessment. 

During his initial psychiatric evaluation, he endorsed depressive and anxiety symptoms including anhedonia, detachment from others, negative self-deprecating ruminations, daily moderate to severe anxiety with frequent panic attacks, intrusive trauma memories, daytime flashbacks, re-experience of his traumatic event, avoidance behaviors, irritability, severe mood swings, hypervigilance within and outside of the home, and chronic insomnia. A comprehensive medical evaluation revealed no alternative underlying medical etiology. He met the DSM-V diagnostic criteria for generalized anxiety disorder (GAD), panic disorder, and PTSD [11].

The patient reported the persistence of these symptoms for multiple years and reported that traumatic triggers and intrusive thoughts increased the severity of his symptoms despite receiving psychiatric and psychological interventions. Prior to his presentation at our clinic, the patient’s treatment history had included a multiple-year course of psychotherapy, as well as engagement in art therapy, active practice of cellular phone-guided mindfulness exercises, physical exercise, dietary optimization, and prior courses of intravenous ketamine infusion therapy. He perceived psychotherapy and psychotropic medications to be ineffective, cellular phone app-guided mindfulness exercises to be partially effective, and intravenous ketamine infusion therapy as effective. His psychotropic medication trials included duloxetine, quetiapine, aripiprazole, haloperidol, lurasidone, lamotrigine, diazepam, lorazepam, propranolol, buspirone, clonidine, hydroxyzine, diphenhydramine, and trazodone. On presentation to this clinic, he was prescribed duloxetine as well as diazepam as needed.

The patient underwent a total of four sets of bilateral DSBs and PRF procedures over a period of 15 months at our clinic. The patient lived out of state and would travel to our clinic to receive a right-sided treatment on the first day and a left-sided treatment the following day. He received one standard DSB treatment (bilateral) and three DSB treatments with PRF (bilateral). In addition to completing a detailed medical and personal history questionnaire, he completed the PTSD Checklist for DSM-V (PCL-5), a 20-item self-report measure that assesses the 20 DSM-V symptoms of PTSD, for the purposes of obtaining baseline measures of PTSD symptom severity. PCL-5 scores above 31 have been shown to be indicative of probable PTSD, and, according to the practice guidelines for PTSD, SGB is indicated for the treatment of PTSD. Prior to his initial treatment, the patient had a PCL-5 score of 73 points, which is far above the clinical threshold of 31 points which is used to give a PTSD diagnosis.

Treatment Series 1: bilateral dual sympathetic blocks

Based on the patient’s symptoms, initial PCL-5 score of 73, and his previous PTSD diagnosis, he underwent bilateral DSB procedures at the C6 and C4 levels. Cervical sympathetic block, or SGB, treatments are generally administered as a part of a multi-part series spanning two appointments one day to one week apart, where the first SGB treatment is performed on the right cervical sympathetic ganglion and the second is performed contralaterally [9]. We performed bilateral DSBs over consecutive days. The patient tolerated the procedure well without any complications and was monitored for 30 minutes in the postop area. During this time, signs of ipsilateral Horner's syndrome (ptosis, miosis, enophthalmos, facial anhidrosis, and conjunctival injection) developed. Signs of Horner's syndrome are taken as evidence of a successful cervical sympathetic blockade. This approach was repeated contralaterally the subsequent day. At the follow-up two weeks after his DSB (bilateral) procedures, the patient reported over 40% improvement in his PTSD symptoms overall, specifically reporting notable relief in terms of his heightened sense of danger. He also reported that he experienced more significant symptom relief from his right-sided SGB treatment and that this symptom relief was more beneficial overall. The patient stated that the severity of his feelings of helplessness had significantly decreased almost immediately after the procedure. Furthermore, the patient’s initial PCL score of 73 dropped to 50 two weeks post-procedure. The benefits of this lasted around three months. Therefore, the patient decided to return to our clinic for further treatment.

Treatment Series 2: bilateral pulsed radiofrequency-enhanced dual sympathetic blocks

Three months following his initial bilateral DSB procedure, he reported anxiety symptom recurrence. At that time, we administered PRF current to his bilateral stellate ganglions at the C6 and C4 levels. 

The patient underwent his first right-sided PRF-enhanced DSB treatment and the procedure was repeated following the same protocol contralaterally at the C6 and C4 sympathetic ganglia the subsequent day. The patient tolerated the procedure without any complications, and signs of ipsilateral Horner's syndrome were noted. The patient denied any negative side effects but reported mild injection site soreness following the procedure. At his two-week post-procedure follow-up visit, he reported significant symptom improvement within two days of undergoing the procedure, with over 80% overall PTSD and general anxiety symptom improvement in the two weeks that followed. His PCL-5 score was 42, compared to his initial pre-procedure score of 73 and the immediate post-procedure score of 50. He reported partial symptom recurrence after two weeks, yet perceived these as tolerable and non-function-impairing, noting overall satisfactory symptom relief that persisted for four months.

Treatment Series 3: bilateral pulsed radiofrequency-enhanced dual sympathetic blocks

The patient returned to our clinic four months after his bilateral PRF-enhanced DSB procedures due to anxiety symptom recurrence. He elected to receive a second series of PRF-enhanced DSBs. The patient underwent his second series of bilateral PRF-enhanced DSB treatments at the C6 and C4 levels over two days. The procedures were well tolerated with no complications and signs of Horner’s syndrome were noted. The procedure was repeated the following day on the contralateral cervical sympathetic ganglion under real-time ultrasonography with an in-plane technique, confirmed by fluoroscopy. At the follow-up, two weeks post-procedure, the patient reported symptom improvement of over 80%, and his PCL-5 score was 22, showing a progressive reduction from 73 (on initial evaluation), 50 (two weeks after bilateral DSB), and 42 (two weeks after bilateral PRF-enhanced DSB). He endorsed persistent symptom relief following this procedure, which lasted for six months.

Treatment Series 4: bilateral pulsed radiofrequency-enhanced dual sympathetic blocks

At six months post-procedure, he presented again due to symptom recurrence. The patient also reported that he continued to take trazodone (100 mg tablet) and diphenhydramine (50 mg/mL injection solution) as needed for managing his insomnia, but noted that his frequency of use decreased significantly after the completion of his bilateral PRF-enhanced DSB. He indicated that although he experienced continued benefits, the degree of symptom relief he felt was less significant compared to the symptom relief he had experienced following his initial DSB treatment series. He had reported over 80% improvement in his PTSD symptoms which had persisted for six months following his last treatment. The patient had started to experience a return of his symptoms in the weeks leading up to this appointment and presented to our clinic to pursue additional treatment.

Over consecutive days, the patient underwent the third and final series of bilateral PRF-enhanced DSB at the C6 and C4 levels. The procedures were well tolerated with no complications and signs of Horner’s syndrome were noted. He reported immediate symptom improvement. At the follow-up, two weeks post-procedure, he reported persistent improvement in anxiety and resolution of depressed mood. His PCL-5 score was further reduced to 20.

Table 1 shows the PCL-5 score changes from baseline and associated time intervals between treatments. A description of the treatment protocol for DSBs and PRF-enhanced DSBs is presented in Table 2.

**Table 1:** PTSD Checklist for DSM-5 (PCL-5) score changes from baseline and associated time intervals between treatments A decrease of 10 points or more is considered a clinically significant response to PTSD treatment. While the patient responded meaningfully to all treatment series, we report that the addition of pulsed radiofrequency in a dual sympathetic blockade of the stellate ganglion (pulsed DSB) sustained significant symptom relief for a longer duration DSB: dual sympathetic blocks; DSM-5: the Diagnostic and Statistical Manual of Mental Disorders, Fifth Edition; PTSD: post-traumatic stress disorder

	Baseline	Two weeks after Treatment Series 1 (bilateral DSB)	Two weeks after Treatment Series 2 (bilateral pulsed DSB)	Two weeks after Treatment Series 3 (bilateral pulsed DSB)	Two weeks after Treatment Series 4 (bilateral pulsed DSB)
PCL-5 score	73	50	42	22	20
Approximate duration of symptom relief	0	3 months	4 months	6 months	6 months

**Table 2:** Description of the treatment protocol for DSBs and PRF-enhanced DSBs We report detailed descriptions of the procedure protocols for bilateral DSB and bilateral pulsed radiofrequency-enhanced DSB that were followed during Treatment Series 1 and Treatment Series 2-4, respectively. Additionally, we report the modifications we have made to the Treatment Series 2 methodology, which now involves bilateral PRF-enhanced DSB treatments with an extended radiofrequency application of 12 minutes in Treatment Series 3 and 4 DSB: Dual sympathetic block; PRF: pulsed radiofrequency

Type and series number of treatment	Description of the treatment protocol
Treatment Series 1: bilateral dual sympathetic block	After preparing the right side of the patient’s neck with povidone-iodine and sterile drapes, a 22 gauge, 2.5 cm needle was directed to the sixth cervical vertebra under real-time ultrasonography with an in-plane technique, placing it in the anterior lateral position, which was also confirmed by fluoroscopy. 7 cc (mL) of bupivacaine 0.5% was injected into and around the sympathetic ganglion at the level of the right C6 anterior tubercle. This methodology was repeated at the fourth cervical vertebra level (C4), with the exception of injecting 3 ccs (mL) of bupivacaine 0.5% into and around the sympathetic ganglion at the level of the right C4 anterior tubercle
Treatment Series 2: pulsed radiofrequency-enhanced dual sympathetic block (six minutes)	The patient’s neck was prepared with povidone-iodine and sterile drapes, and the skin was anesthetized. Using ultrasound guidance, a 20 gauge, 100 mm insulated needle with a 10 mm, 42 °C active tip was directed to the sixth cervical vertebra, placing it in the anterior lateral position, which was confirmed by fluoroscopy. A 2 Hz current with a maximum voltage limit of 45 V was applied for a total of six minutes. After administering the radiofrequency current, 7 mL of bupivacaine 0.5% was injected into and around the C4 stellate ganglions and 3 mL of bupivacaine 0.5% was injected into and around the C6 stellate ganglion
Treatment Series 3 and 4: pulsed radiofrequency-enhanced dual sympathetic block (12 minutes)	The patient underwent bilateral PRF-enhanced DSB treatments at the C6 and C4 levels over two days according to the treatment protocol described for Treatment Series 2, with the exception of the pulsed radiofrequency current administered for 12 minutes (vs. the applied current of six minutes in Treatment Series 2)

## Discussion

This is the first case report involving a patient who was followed up over one year with successful management of his PTSD symptoms by using PRF at increasing time intervals of three months, four months, and six months between procedures. SGBs, dual blocks in particular, have become more accessible within select treatment settings for the treatment of PTSD, as the general public has progressively gained awareness of its clinical utility. In our experience of treating PTSD and severe anxiety patients with DSBs by using a local anesthetic, we have found that while significant symptom improvement is achieved post-procedurally, the durability of this improvement has limited the wider adoption of this treatment modality. Therefore, we have adopted PRF ablation following successful local anesthetic sympathetic blocks. In our patient, the first PRF-enhanced DSB, which was performed over six minutes, provided clinically significant symptom relief that persisted for four months. The second PRF-enhanced DSB was performed over 12 minutes and yielded symptom relief for six months. We also highlight a progressive 53-point reduction in PCL-5 scores throughout the treatment course. This suggests that the efficacy of PRF-enhanced DSB may be additive over successive procedures.

Since PRF in SGB therapy is a relatively recent development in PTSD treatment research, the mechanism of action for extended symptom relief remains unclear. Nonetheless, a literature search revealed one published report on the use of SGB with PRF in PTSD [12]. This case report by Lipov describes selective blockade of the stellate ganglion with the addition of PRF in a patient with PTSD and the results elucidate possible physiological mechanisms that may explain this development [12]. Among these, the hypothesis that DSB with PRF may reset neuronal circuitry that connects the sympathetic nervous system with important brain regions responsible for coordinating defense mechanisms and emotional processing has been further supported in recent literature [7,9,13]. Lipov also highlights previously published evidence that PRF leads to the upregulation of c-Fos, a marker of neuronal activity, and PRF-mediated activation of c-Fos in specific brain centers such as the dorsal horn has been shown to persist for several days after the current is applied, suggesting a possible explanation for the extended benefits of PRF in DSB therapy [12].

Despite the encouraging results from this case report, further investigation of PRF-enhanced DSB is warranted to deepen our understanding of the physiological mechanisms and consequences of the addition of PRF to DSB and to standardize this intervention protocol. Questions remain as to what the ideal duration of time is in terms of delivering PRF current, and we note that further research is needed to explore this point.

## Conclusions

Although the traditional DSB procedure has a strong safety and efficacy profile, a subset of PTSD patients may not achieve sufficient or durable symptom relief from it. This case study highlights the enhanced efficacy of DSB with the addition of PRF in one PTSD patient, as measured by a progressive reduction in PCL-5 scores throughout the treatment course. Furthermore, our results suggest that modifying the traditional DSB procedure to include PRF extended the duration of symptom relief in this PTSD patient. The results from this case study warrant further investigation into the safety and clinical efficacy of PRF-enhanced DSBs for PTSD patients with limited symptom relief from the traditional DSB treatment modality. Moreover, further studies investigating if the addition of PRF to DSB could be useful for clinical applications other than PTSD, as these therapies are used in the treatment of various health conditions. In our patient's own words, the PRF-enhanced DSBs have been “life-changing” for him, when traditional psychiatric treatments failed to provide the desired outcomes.
